# Environmental Particulate Matter and Genetic Alterations: Tarantini et al. Respond

**DOI:** 10.1289/ehp.0900830R

**Published:** 2009-08

**Authors:** Letizia Tarantini, Matteo Bonzini, Valeria Pegoraro, Valentina Bollati, Barbara Marinelli, Laura Cantone, Giovanna Rizzo, Pier Alberto Bertazzi, Andrea Baccarelli, Pietro Apostoli, Lifang Hou, Joel Schwartz

**Affiliations:** Laboratory of Environmental Epigenetics, Department of Preventive Medicine and Department of Environmental and Occupational Health, University of Milan and IRCCS, Maggiore Hospital, Mangiagalli and Regina Elena Foundation, Milan, Italy, E-mail: letizia.tarantini@unimi.it; Department of Experimental and Applied Medicine, Occupational Medicine and Industrial Hygiene, University of Brescia, Brescia, Italy; Department of Preventive Medicine, Feinberg School of Medicine, Northwestern University, Chicago, Illinois, USA; Exposure, Epidemiology and Risk Program, Department of Environmental Health, Harvard School of Public Health, Boston, Massachusetts, USA

We thank Cetta et al. for the interest they express in our recent article (Tarantini et al. 2009). The basis for our hypothesis that foundry workers exposed to air particles might have hypomethylation of DNA repetitive element was existing evidence demonstrating that inhaled airborne particles induce oxidative stress and *in vitro* studies indicating that oxidative stress might generate hypomethylation throughout the genome. We are glad to see that Cetta et al. also find such hypothesis well grounded in previous existing work. Our investigation showed for the first time that airborne particles induce hypomethylation in repetitive sequences that are widely represented across the human genome, and indicated that hypomethylation of the inducible nitric oxide synthase (*iNOS*) gene is one potential mechanism contributing to particle-induced oxidative stress and inflammation. In addition, we showed that such processes can be detected in a DNA source, such as peripheral blood leukocytes, which is easily obtainable from human subjects. Our findings might be extended to ambient air pollution exposures, as suggested by our related investigations demonstrating repetitive element hypomethylation, as well as other gene-specific modifications, in blood DNA from individuals exposed to airborne benzene (Bollati et al. 2007) or to ambient particulate matter (Baccarelli et al. 2009).

In their letter, Cetta et al. rightly emphasize the possible roles of personal genetic features in determining which individuals will develop adverse health outcomes in response to air particle exposure. This is also confirmed by several other investigations that evaluated different health-related end points, including our previous work demonstrating genetic polymorphisms in pathways related to oxidative stress responses (Chahine et al. 2007) and our results on methyl nutrient metabolism (Baccarelli et al. 2008), both of which augment the negative effects of ambient particulate matter on cardiac autonomic function. In comparison with genetic variations, DNA methylation and other epigenetic modifications are of particular interest with respect to air particle effects, because—as demonstrated by animal models of environmental epigenetic toxicity —they are reversible and may be restored to their original state by dietary or pharmacologic interventions (Baccarelli and Bollati 2009; Dolinoy and Jirtle 2008; Jirtle and Skinner 2007).

We agree that the investigation of mechanisms closer to the final health outcomes in the chain of events started and/or maintained by air pollution exposures is as important as investigating early events. Studies tackling air pollution effects from different angles are needed to link together early events with the diseases of concern. As early events may be more susceptible to interventions aimed at reversing them or slowing their progression, we hope that our contribution to identify novel modifiable mechanisms induced by air particle exposures can help reach the goal recently set forth for environmental scientists by the newly appointed director of the National Institute of Environmental Health Sciences “to prevent or stop the progression of complex health problems” (Birnbaum 2009).
